# Computational Study of Quasi-2D Liquid State in Free Standing Platinum, Silver, Gold, and Copper Monolayers ^†^

**DOI:** 10.3390/condmat1010001

**Published:** 2016-03-02

**Authors:** Li-Ming Yang, Ariel B. Ganz, Matthew Dornfeld, Eric Ganz

**Affiliations:** 1 School of Chemistry and Chemical Engineering, Huazhong University of Science and Technology, Wuhan 430074, China; 2 Division of Nutritional Sciences, Cornell University, 339 Savage Hall, Ithaca, NY 14853, USA; 3 Department of Physics, University of Minnesota, 115 Union St., SE, Minneapolis, MN 55416, USA

**Keywords:** 2-D melting, molecular dynamics, density functional theory, 2-D liquid

## Abstract

Recently, freestanding atomically thick Fe metal patches up to 10 atoms wide have been fabricated experimentally in tiny pores in graphene. This concept can be extended conceptually to extended freestanding monolayers. We have therefore performed *ab initio* molecular dynamics simulations to evaluate the early melting stages of platinum, silver, gold, and copper freestanding metal monolayers. Our calculations show that all four freestanding monolayers will form quasi-2D liquid layers with significant out-of-plane motion and diffusion in the plane. Remarkably, we observe a 4% reduction in the Pt most likely bond length as the system enters the liquid state at 2400 K (and a lower effective spring constant), compared to the system at 1200 and 1800 K. We attribute this to the reduced average number of bonds per atom in the Pt liquid state. We used the highly accurate and reliable Density Functional Theory (DFT-D) method that includes dispersion corrections. These liquid states are found at temperatures of 2400 K, 1050 K, 1600 K, and 1400 K for platinum, silver, gold, and copper respectively. The pair correlation function drops in the liquid state, while the bond orientation order parameter is reduced to a lesser degree. Movies of the simulations can be viewed online (see Supplementary Material).

## 1. Introduction

Historically, it has been difficult to fabricate freestanding monolayer metal films experimentally. However, recently, freestanding atomically thick iron membranes suspended in and supported by tiny graphene pores have been fabricated [1]. These iron layers were only up to 10 atoms wide. This is an exciting development, but due to the presence of the pores around the periphery, the iron patches differed structurally from the predictions for an extended freestanding 2D monolayer. This finding demonstrates the potential of perforated graphene as a support for small 2D monolayers, and paves the way for novel freestanding 2D structures to be formed. Zhao *et al.* used density functional theory (DFT) calculations to study these systems, and predicted that the largest thermodynamically stable patch would be 12 atoms across [1]. In our paper, we will be studying freestanding systems of quasi-2D metal atoms with significant out-of-plane motions, using highly accurate Density Functional Theory with dispersion corrections (DFT-D).

The use of small pores in graphene to support metals could potentially be used to fabricate different metal patches or small freestanding metal monolayers. Following the discovery of iron patches in graphene pores, this method has been extended computationally to small gold patches in the pores [2]. Koskinen and Korhonen (K & K) studied the solid and liquid phases of a small monolayer gold patch in a graphene hole using primarily the less reliable density-functional tight-binding (DFTB) method [2]. This 49-atom gold patch stayed solid up to 700 K, and then at 900 K formed an unusual quasi-2D liquid layer. In fact, K & K also extended this study to a close packed quasi-2D monolayer of gold in a periodic cell. They also checked this result using more reliable DFT. Remarkably, they observed quasi-2D liquid behavior, and predicted a melting point of 1200 K.

In recent years, there has been tremendous interest in two-dimensional materials due to their intriguing properties and potential applications in nanoscience and nanotechnology [3]. 2D materials have a much larger surface area compared to the bulk phase, leading to unique thermal, electrical, and mechanical properties. Melting of 2D materials is substantially different than melting in the bulk. For example, in three dimensions, theoretically one can define the melting point as the boundary between the Gibbs free energy minima of the solid and liquid phases. For two dimensions, various criteria have been proposed. A modified Lindemann criterion for use in 2D was proposed by Zheng *et al.* [4].

More recently, a dynamic criteria for melting in two dimensions was proposed by Zahn and Maret [5]. Freestanding 2D sheets exhibit significant motion out of the plane in addition to the introduction of defects and long range corrugations as the temperature is raised. In this paper, we will refer to systems with significant out-of-plane motion as quasi-2D. Zakharchenko *et al.* have studied the high temperature behavior of graphene using atomistic simulations [6], and Singh *et al.* have studied the vibrational stability and melting behavior of freestanding MoS_2_ [7]. Los *et al.* predict a more accurate melting point of T_m_ = 4510 K for graphene using nucleation theory, which is about 250 K higher than that of graphite, and so far the highest of all materials [8]. A melting point of 3700 K has been predicted for single layer freestanding MoS_2_ [7]. In 2015, Merino and coworkers predicted the melting of boron 40 molecules in DFT simulations. They consider this system to be a nanobubble, and observed diffusion of individual atoms in the shell [9].

There is a long history of the study of melting in two dimensions. In the 1970s, the Kosterlitz–Thouless theory was developed [10]. According to the Kosterlitz–Thouless–Halperin–Nelson–Young (KTHNY) theory, the melting mechanism of 2D crystals proceeds via two consecutive continuous transitions, caused by the creation of topological defects [10–12]. One characteristic of the melting of many 2D systems is the existence of a distinct hexatic phase. This phase shows quasi-long-range bond orientational order, even as the positional order becomes short range. A recent study of 2D hard disks found unusual two-stage melting [13]. A follow up study on quasi-2D hard spheres with significant out-of-plane particle motions [14] found that the peculiar two-stage melting scenario of a continuous solid-hexatic and a first-order hexatic–liquid transition, as observed for a truly 2D system of hard disks, persists [13]. Dijkstra and coworkers found that the hexatic phase was only observed in a narrow range of parameters [14].

The individual bond strength of these freestanding 2D metal layers at 0 K is larger than in the bulk due to the reduction in total bonds per atom (6 *versus* 12). For Ag, the individual bond strength actually increases from 0.42 eV for the bulk to 0.66 eV for the freestanding 2D layer [15]. The 2D Ag monolayer and the 3D bulk have cohesive energies of 2.01 and 2.49 eV atom, respectively [15]. We have also studied the properties of the freestanding Au monolayer [16]. We observe an increase in bond strength from 0.52 eV/bond for the 3D bulk gold to 0.94 for the freestanding 2D monolayer [16]. The increased bond strength of 2D layers also explains why the melting point of graphene is higher than that of diamond [8].

The melting points of the bulk materials correlate with the bond strengths of these materials. For example, the bulk Cu, Ag, Au, and Pt cohesive energies are 3.48, 2.49, 3.11, and 5.84 eV, respectively [17]. We see that platinum has the highest cohesive energy. The bulk melting points are 1356, 1235, 1338, and 2041 K, respectively [18]. We expect that the temperatures of the liquid states or melting points of the freestanding quasi-2D monolayers will also scale with cohesive energy and bond strength. We see that platinum also has the highest bulk melting point. Therefore, we can expect that the freestanding 2D platinum monolayer should be the most stable of the four materials. Note that bulk platinum is also a noble metal, and therefore the 2D platinum monolayer may be relatively durable and resistant and thus more favorable for experimental fabrication.

In this paper, we use *ab initio* molecular dynamics simulations to study freestanding quasi-2D monolayers of platinum, silver, gold, and copper. We will use freestanding periodic 8 × 8 simulations, the results of which can be applied to a broad range of situations. Furthermore, the isolated freestanding monolayer is of fundamental interest. Real experimental materials will, of course, need to be supported in some way. For example, either a monolayer could be grown on a substrate, which is then etched away underneath to leave a freestanding section, or a freestanding monolayer could be transferred onto a substrate over a small hole. Alternatively, metal atoms could be added to a graphene layer with tiny holes to form embedded patches as has been demonstrated by Zhao *et al.* [1]. We believe that our results should have general application to a variety of situations, and contribute beyond the existing studies of tiny patches embedded holes in graphene.

We use the DFT-D method, which includes dispersion corrections that are necessary for the long bond distances found in these systems. We calculated the diffusion and particle correlation functions to demonstrate that the layers are liquid at specific temperatures. Analysis of bond numbers and bond lengths revealed unexpected results with the platinum layer showing unusually low bond lengths in the liquid state. The pair correlation function drops in the liquid state, while orientational order is reduced to a lesser degree. Movies of the simulations can be viewed online.

## 2. Computational Methods

**Figure 1:**
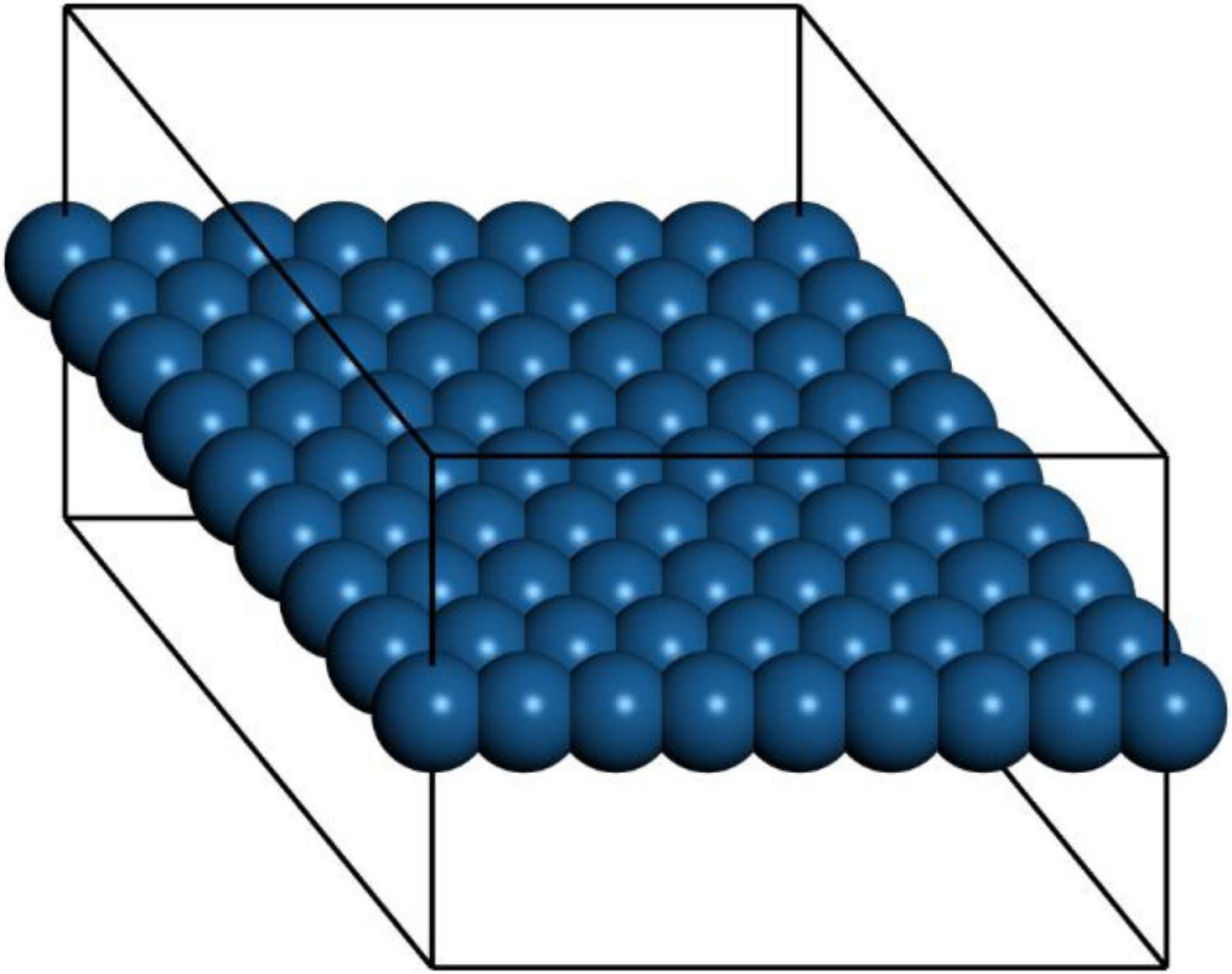
Single unit cell of freestanding 8 × 8 Pt model at 0 K.

*Ab initio* Born–Oppenheimer molecular dynamics simulations were performed. A periodic boundary condition unit cell that holds an 8 × 8 array of atoms (21.0 × 21.0 × 15.0 Å for Pt) was used. One unit cell in the X–Y plane along with the outline of the periodic cell are shown in Figure 1. The 15-Å spacing in Z between layers ensures good isolation in each layer. For Cu, Ag, and Au, the edge dimensions of the unit cells were 19.3 Å, 22.3 Å, and 21.86 Å, respectively. The scalar-relativistic DFT-D and Tkatchenko–Scheffler (TS) method were used in CASTEP [19] in Materials Studio 7 and 8. Molecular Dynamics (MD) simulation in the constant number, volume, temperature (NVT) ensemble were carried out for 6–27 ps with a time step of 2.5 fs for gold and copper, and 4 fs for silver and platinum. The parameters were: Accuracy = fine; Self-Consistent Field (SCF) = 3 × 10^−6^; Smearing = 0.04; Direct Inversion of the Iterative Subspace method (DIIS) = 20; Nosé–Hoover method [20], Nosé Q = 2, Nosé chain length = 2; Fixed center of mass. For the higher temperatures, the first 12 ps of each run were considered an equilibration period (less time was needed for the lower temperatures). More details on each run are in the Supplementary Information (SI). Data were acquired after the equilibration. Materials Studio was also used to create the initial structures and visualize the results. The Root Mean Square Displacement (RMSD) was calculated using the differences between an initial data frame (after equilibration), and the final frame of each simulation.

The pair correlation function for our quasi-2D system *g(r)* was calculated, where σ is the two-dimensional atomic density, N is the total number of atoms, and *δ* is the delta function.

(1) $$g\left(r\right)=\frac{1}{2\pi r\sigma N}\langle \sum_{i}\sum_{j\neq i}\delta \left(r-r_{ij}\right)\rangle$$

The large vacuum space between layers assures good isolation of the freestanding quasi-2D layer. The diffusion coefficient D was calculated using < *r*^2^ > = 4*Dt*. The R computing environment [21] and Python programming environment [22] were used for plot generation and data analysis. The RMSD plots at higher temperatures were fit to $y=\sqrt{a+bt}$, to include both Z motion and X–Y diffusion. The Forcite analysis program was used to generate velocity correlation functions in Materials Studio. Origin 2015 was also used to make some plots. The bond orientation correlation function was calculated by locating the nearest neighbors for each atom in each frame (atoms within 1.38 times the distance P = 2.76, 3.06, 2.88, and 2.56 Å, respectively, for copper, silver, gold, and platinum). The 2D bond angle was then determined by ignoring the Z coordinate of each atom, and then determining the angle compared to the X-axis. Each bond centroid was calculated. Then, a modified distribution function *g*6(*r*) = < *cos* (6*θ*) >, where *θ* is the difference between two bond angles, and *r* is the full 3D distance between two bond centroids was calculated. The factor of six ensures that six-fold symmetry of the perfect close packed layer will produce a result of one.

The bond length distributions have been fit to a Boltzmann distribution shown in Equation (2). We approximate the energy the E of bond length deviation $\left(\ell -\ell_{0}\right)$ using a simple spring model with spring constant k_spring_ at low deviations, as shown in Equation (3). Here, ℓ is the bond length, and $\ell_{0}$ is the most probable bond length at a given temperature, and corresponds to zero spring extension. We have also included a hard sphere repulsive term ${\left[\ell -r_{0}\right]}^{12}$ with C and r_0_ determined empirically for each plot.

(2) $$P=Ae^{-E∕k_{B}T}$$

(3) $$E=\frac{1}{2}k_{spring}{\left[\ell -\ell_{0}\right]}^{2}+C∕{\left[\ell -r_{0}\right]}^{12}.$$

## 3. Results and Discussion

We performed a set of *ab initio* Born–Oppenheimer molecular dynamics simulations to study the melting properties of freestanding quasi-2D Pt, Ag, Au, and Cu monolayers. The results are organized as follows. For all of the systems at low temperatures, the system maintains the framework during the simulations. Long wavelength oscillations are observed in side views, and then larger amplitudes and shorter wavelength oscillations are observed as the temperatures are raised (snapshots showing side views are included in the SI).

At intermediate temperatures, larger oscillations in the Z direction were observed as well as substantial bond length extensions; however, the atoms oscillate back and forth without any net diffusive motion or atomic switching. The hexagonal close pack (HCP) arrangement is maintained and each atom has six bonds. In this temperature range, the bond length extensions are very large, ranging from 20%–36%. We note that such large bond length extensions could be associated with bulk melting at real world time scales and in experimental conditions. However, the freestanding quasi-2D metal films we are discussing in this paper are a very different case, and must be studied independently.

Finally, at higher temperatures, we observe the beginnings of diffusive motion, and atom switching. It is in this temperature range that we observe quasi-2D melting behavior (we will call this the quasi-2D liquid regime). In some cases, we see individual adatoms on top of the layer. Each material is discussed below in detail, with a focus on the quasi-2D melting and quasi-2D liquid behavior below. If we raise the temperature beyond this regime, the monolayers become unstable, and we see larger 3D structures forming leaving behind large holes.

### 3.1. Platinum

**Figure 2:**
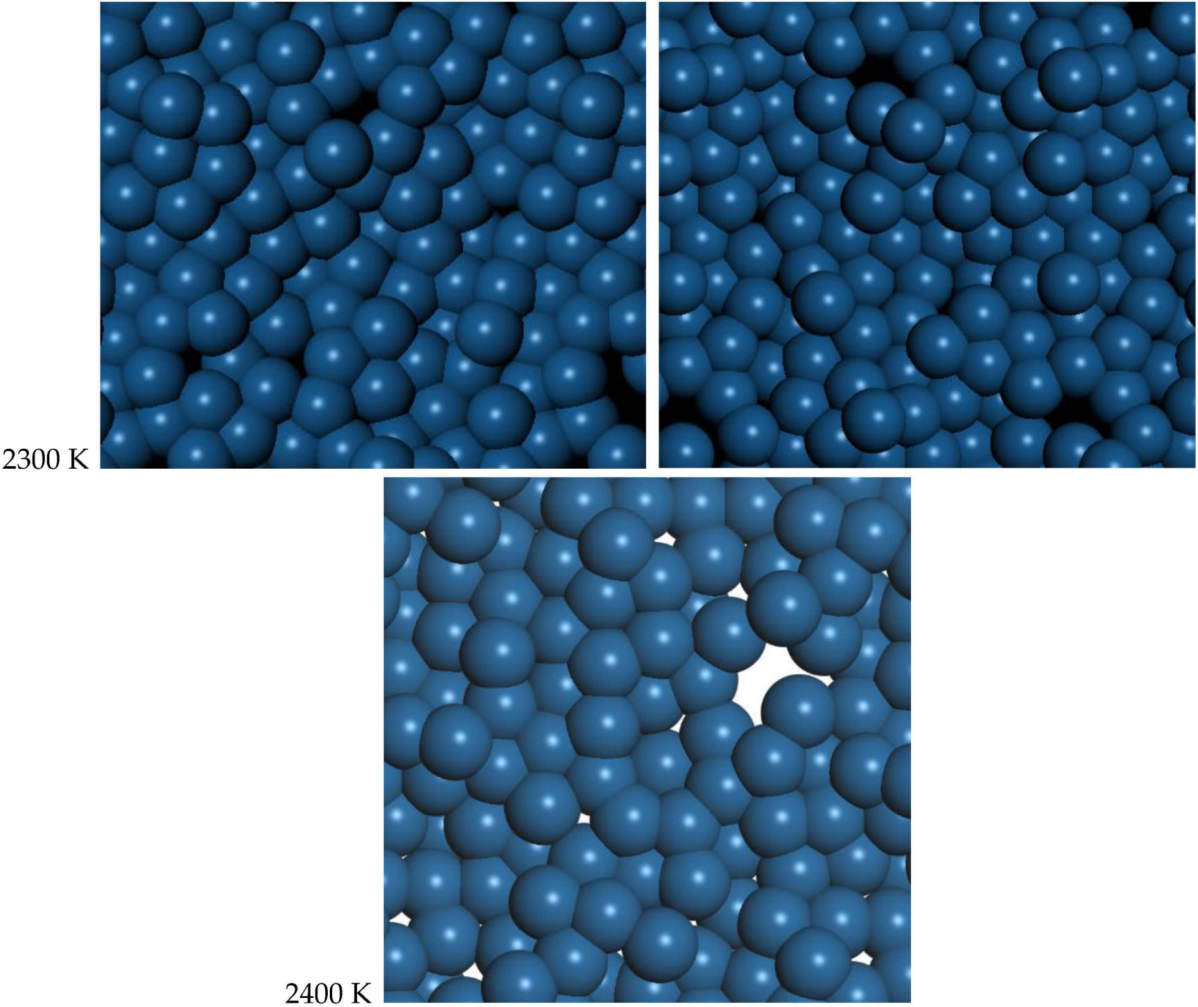
Molecular dynamics snapshots of quasi-2D Pt layer. We show two snapshots at 2300 K, separated by 0.2 ps and one snapshot at 2400 K. Atoms are mobile and holes come and go. This is quasi-2D liquid behavior.

The range from 2300 K to 2400 K represents the quasi-2D liquid regime for platinum. At 2300 K, we see an increasing number of small holes, and the atoms are now able to diffuse. At 2400 K, we see increasing amounts of diffusion, and the system has larger holes, and more excursions in the Z direction. Snapshots from the simulation are shown in Figure 2. A ball and stick model is also shown in the SI to highlight the binding configuration and holes. A movie of the 2400 K Pt simulation is shown in the SI, and one can see that the atoms are moving vigorously.

**Figure 3:**
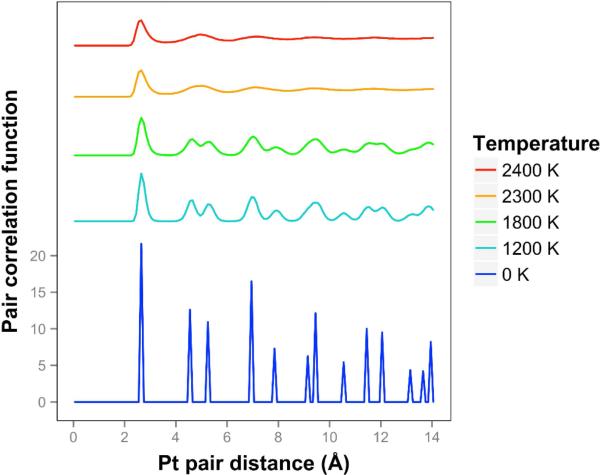
Pair correlation function for freestanding platinum quasi-2D monolayer at temperatures ranging from 0 K to 2400 K. At 2300 K and 2400 K the pair correlation function drops off quickly, and the freestanding quasi-2D Pt monolayer is a quasi-2D liquid. Higher temperatures are offset for visibility.

We see that the pair correlation function for the quasi-2D Pt layer drops away quickly at 2300 and 2400 K (see Figure 3). We see that the two next nearest neighbor peaks near 5 Å merge together at 2300 K and above. For lower temperatures, at and below 2200 K, the framework is maintained, although there is substantial motion in the Z direction and substantial bond length extension.

**Figure 4:**
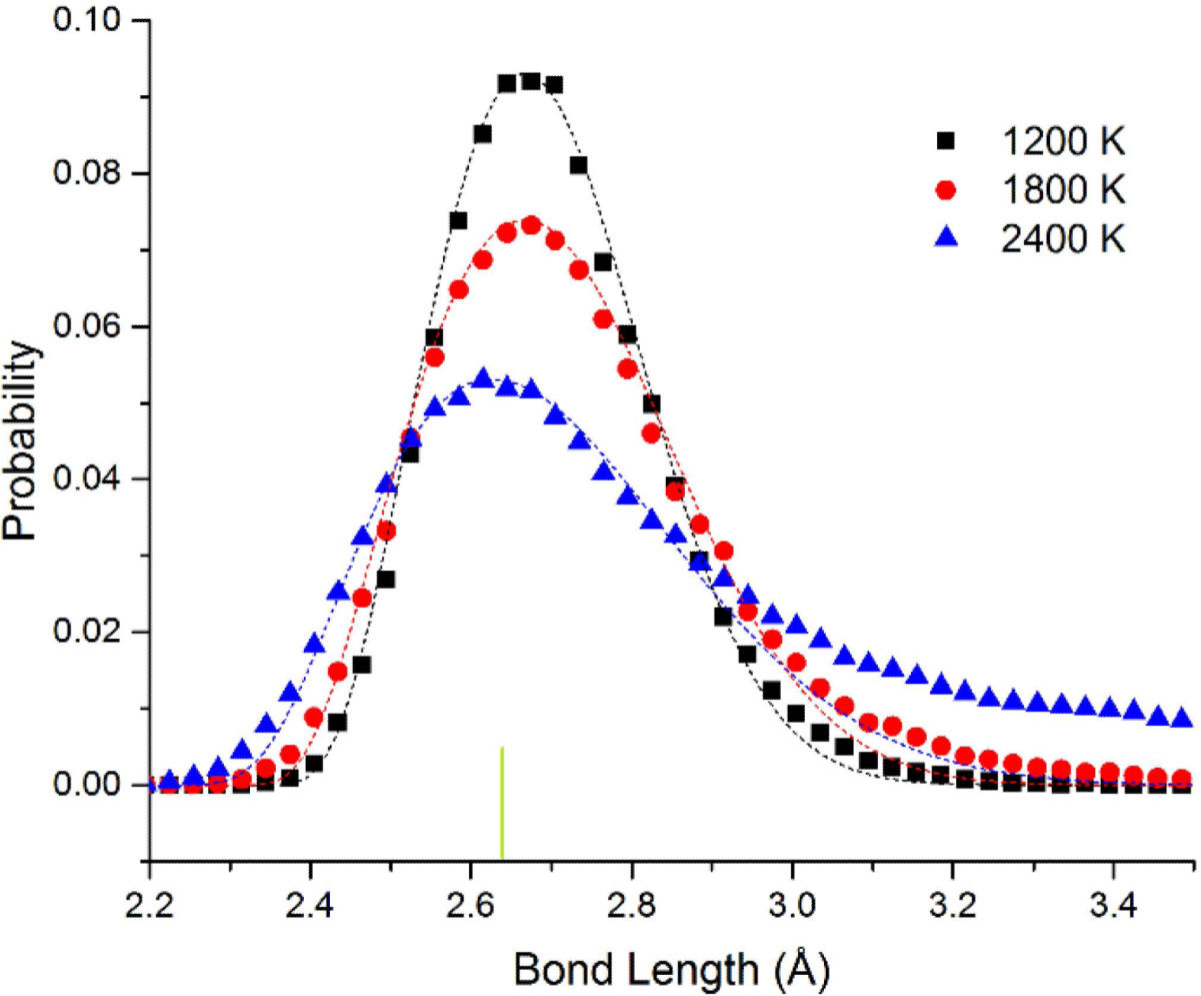
Bond length distribution for freestanding quasi-2D Pt monolayer at 1200 K, 1800 K, and 2400 K. The 0 K bond length of 2.63 Å is shown as a short vertical green line below the curves. At 1200 K and 1800 K, almost all of the bond lengths are well above the 0 K value. The most likely length shrinks by 4% at 2400 K in the liquid state, compared to 1200 and 1800 K. Solid lines are fitted to the Boltzmann distribution as discussed in the text.

In Figure 4, we show the bond length distribution for freestanding quasi-2D Pt monolayer at 1200 K, 1800 K, and 2400 K. At 1200 K and 1800 K, the framework is maintained, but extensive motion is observed in the Z direction. The bond length distribution is almost all well above the 0 K bond length of 2.63 Å (shown as the short vertical line below the curves). However, at 2400 K in the liquid state, due to the presence of holes, the average number of bonds per atom is reduced from 6 to 5.1. This can also be seen clearly in the ball and stick model at this temperature shown in the SI.

**Figure 5:**
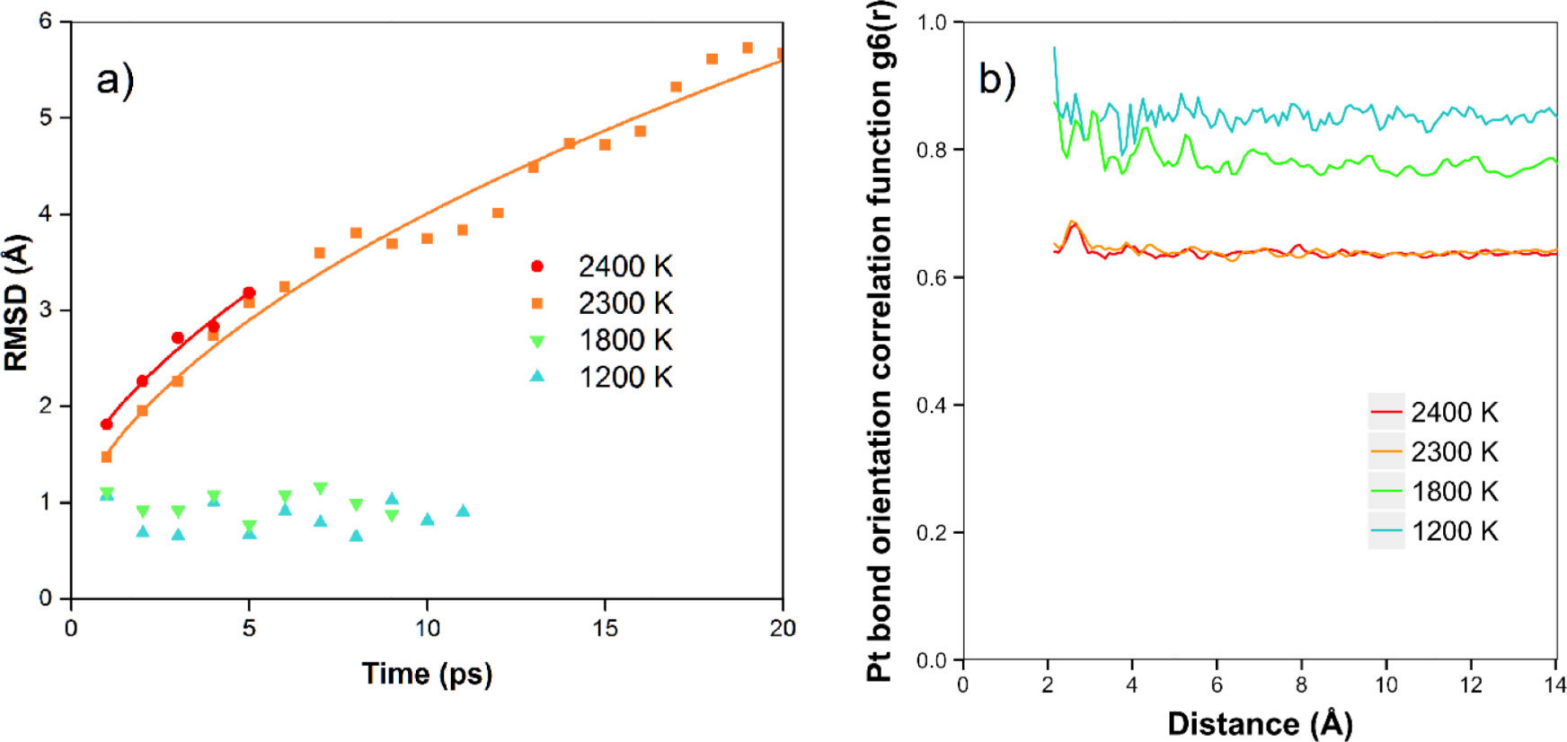
(**a**) Root mean square displacement (RMSD, in Å) for platinum simulations at 1200–2400 K. At lower temperatures, the RMSD is constant around 1 Å due to oscillation primarily in the Z direction. At 2300 and 2400 K, we see extended diffusion. (**b**) Bond orientation correlation function.

In Figure 5a, we show the results of the RMSD calculations for the platinum simulations. We see that at the lower temperatures of 1200 K and 1800 K, there is no net diffusion, and the RMSD has a relatively constant value of approximately 1 Å over times of 8–12 ps. In this case, we expect the RMSD to be mainly due to the motion of the atoms moving in the Z direction, with amplitude dependent on temperature, although there is no net diffusion in the plane yet during the simulation times. At higher temperatures (2300 K and 2400 K), where the atoms are starting to diffuse and are more free to move to different locations (in the quasi-2D liquid regime), we see that the RMSD is increasing with time. In these cases, at longer times we observe the expected 2D random walk diffusion characteristic. At the higher temperatures of 2300 and 2400 K, where we are in the quasi-2D liquid regime, we observe net diffusion consistent with random walk behavior. We find RMSD values of 5.7 Å and 3.2 Å after 20 and 5 ps, respectively. We can estimate the diffusion coefficient D = 0.4 Å^2^/ps for 2300 K, and 0.5 Å^2^/ps for 2400 K. The velocity auto correlation function for Pt at 2400 K is shown in the SI. We observe that the correlation dissipates quickly after less than 1 ps. In Figure 5b, we also show the bond orientation order parameter for platinum. We see that this is close to one for short distances and lower temperatures, and is reduced as we raise the temperature. Although the value drops somewhat with distance, we see that bond orientational order is maintained over the longest distance available in the simulation. For 2400 K, where we are in the liquid state, we see an average value of around 0.63–0.67.

The data points have been fitted to the Boltzmann distribution shown in Equation (2). We approximate the energy *E* of the bond length deviation (d–d_0_) using a simple spring model with spring constant k_spring_ at low deviations as shown in Equation (3) (with an additional hard sphere repulsive term). For Pt, we see that the effective spring constant k_spring_ = 4.3 and 4.1 eV/Å^2^ at 1200 and 1800 K. The fits are quite good at all temperatures. At 2400 K, the system changes to the liquid state. This state has 4% shorter bond length at the maximum. We attribute the shortened bond lengths to the lower number of bonds in the liquid state. We observe that the effective spring constant decreases to k_spring_ = 2.8 eV/Å^2^. This shrinking as we raise the temperature corresponds to a negative coefficient of thermal expansion as we enter the liquid state. at 2400 K, at long bond lengths, there are significant excess observations from 2.8 to 3.5 Å. This is expected as we approach the van der Waals limit as the atom moves away from the plane of the other atoms (the bonding becomes weaker than the spring model and more atoms will be observed with these long bond lengths).

We also explored the effect of raising the simulation size to 10 × 10 atoms for the plantinum model. A single run at 2150 K for 21 ps gave similar results to the 8 × 8 models at 2300–2400 K.

### 3.2. Gold

**Figure 6:**
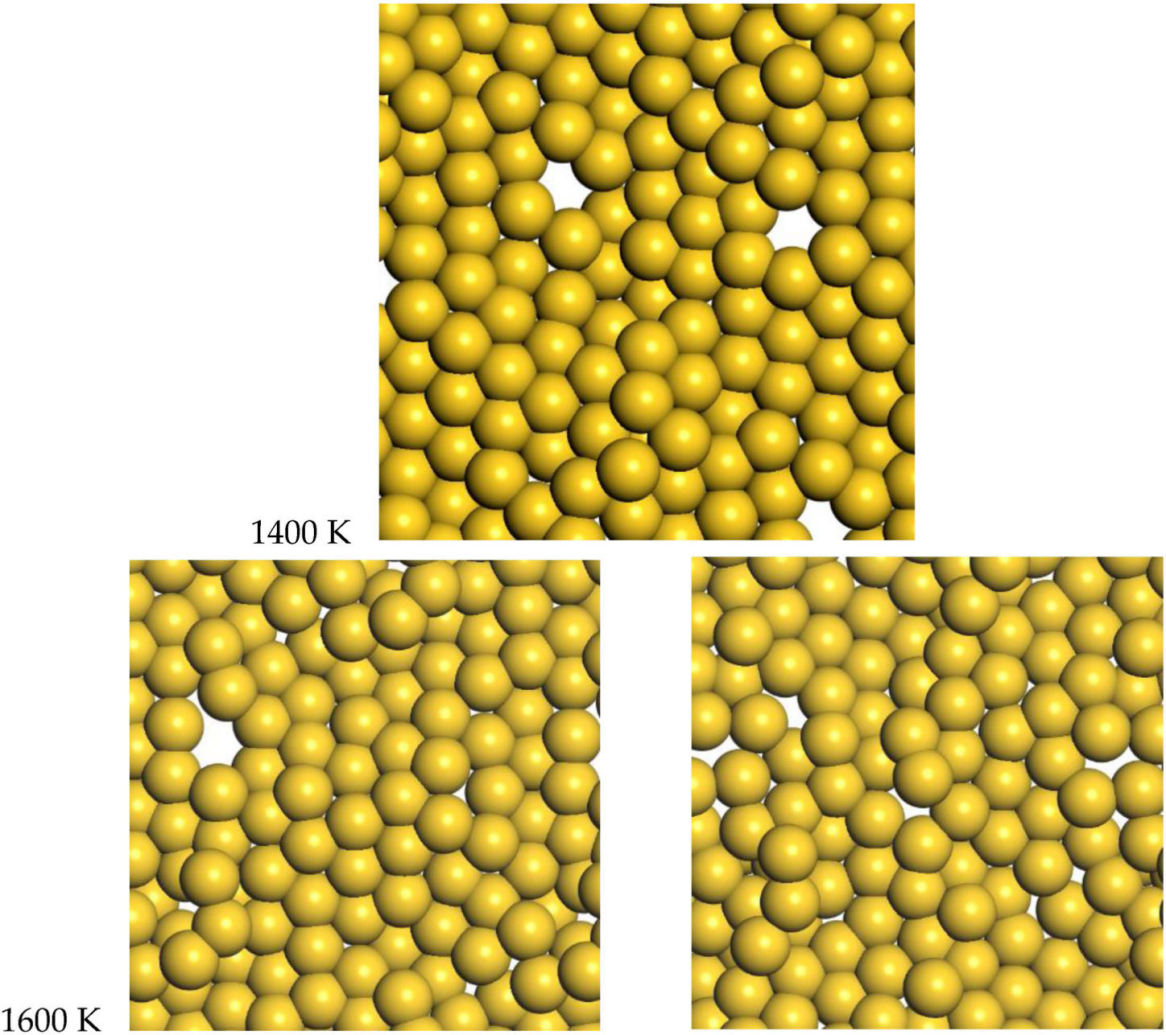
Molecular dynamics snapshots of quasi-2D Au layers at 1400 K and 1600 K. At 1600 K, we show two frames separated by 0.5 ps. quasi-2D liquid behavior is observed at these temperatures.

For gold, the quasi-2D liquid regime is 1400–1600 K. For this range of temperatures, we see the quasi-2D layer is maintained, but also occasional small holes and adatoms are observed. Snapshots from the molecular dynamics simulations at 1400 K and 1600 K are shown in Figure 6. A movie of the 1600 K Au simulation is shown in the SI. At 1400 K and 1600 K, diffusion is observed, with typical RMSD = 2.4 Å and 3.4 Å after 12 ps, respectively (see Figure S7). These displacements give diffusion coefficients of D = 0.12 and 0.25 Å^2^/ps, respectively. These can be compared to the result of 0.14 Å^2^/ps at 1600 K from K & K [2]. At 1600 K, the average number of bonds is 5.5, somewhat reduced compared to the low temperature results, but still larger than the Pt results at 2400 K.

We also raised the temperature to 2000 K, but at this temperature the gold layer is unstable and falls apart. Large holes are left behind and nanowires or nanobridges form. A snapshot from the end of the simulation is shown in the Supplementary Information (SI).

**Figure 7:**
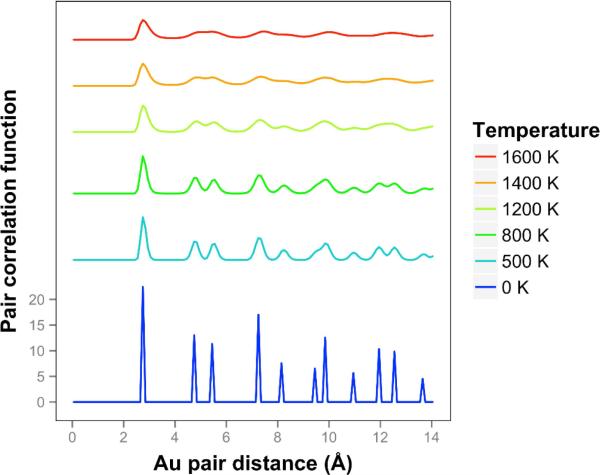
Pair correlation function for gold quasi-2D layer at temperatures ranging from 0 K to 1600 K. Higher temperatures are offset for visibility.

Looking at the pair correlation function for the quasi-2D gold layers (shown in Figure 7), we observe that the correlation is significantly reduced as the temperature is increased. At longer distances, the correlation flattens out and drops away. The two next nearest neighbor peaks near 5 Å merge together at temperatures of 1400 K and above. We can compare our correlation results to the previous results of K & K [2]. The shapes of the curves are quite different, and it should be noted that they are using the less accurate DFTB theory. They identified a melting point of 1200 K. Considering the disappearance of the two next nearest neighbor lines and the general disappearance at longer distances, a comparable temperature for our results would be 1400 K. By including the diffusive interactions (which are very important for our system with such long bond length extensions and in the liquid regime), and using a more accurate DFT-D theory, we see a substantial 20% increase in temperature compared to the K & K results. Note that K & K also did DFT calculations on these Au models and included movies but not correlation functions of the results.

The cell dimensions were set using 0 K optimization. We tested the thermal expansion from 0 K up to the liquid state temperatures (this question was also explored by K & K for gold). We find a roughly 3% 2D areal expansion leading to a 1.7% linear change in dimensions. We did run some simulations with a 1.7% linearly expanded cell, but the results were unchanged. The large Z motions dominated these simulations; thus, a small 1.7% linear change in cell dimension is not significant. An insignificant amount of stress is imposed as the atoms are moving back and forth in the Z direction.

### 3.3. Silver

**Figure 8:**
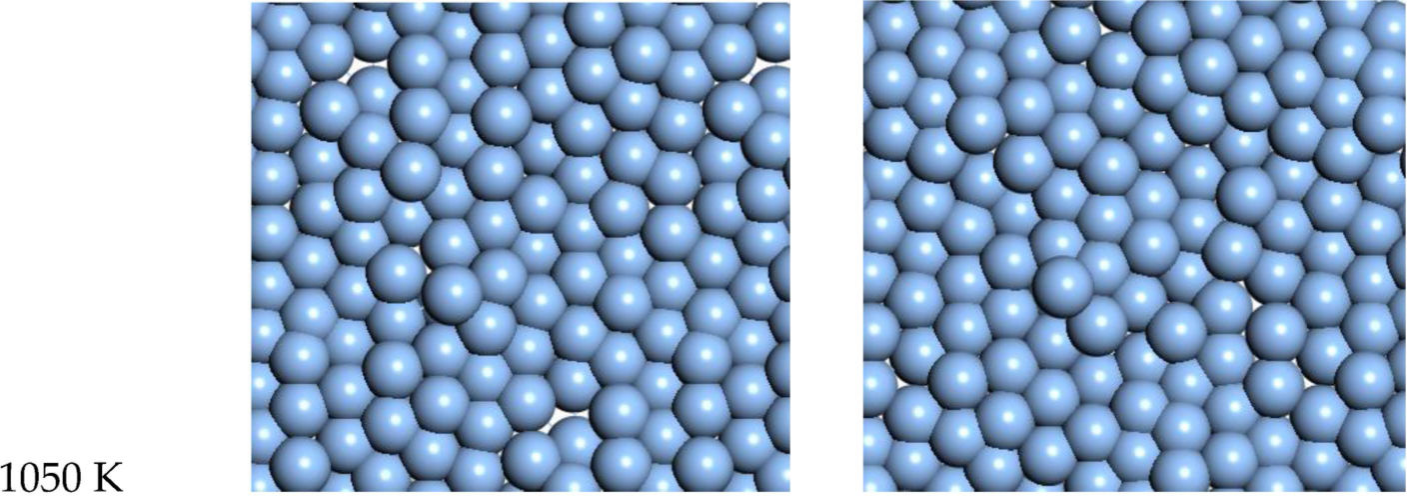

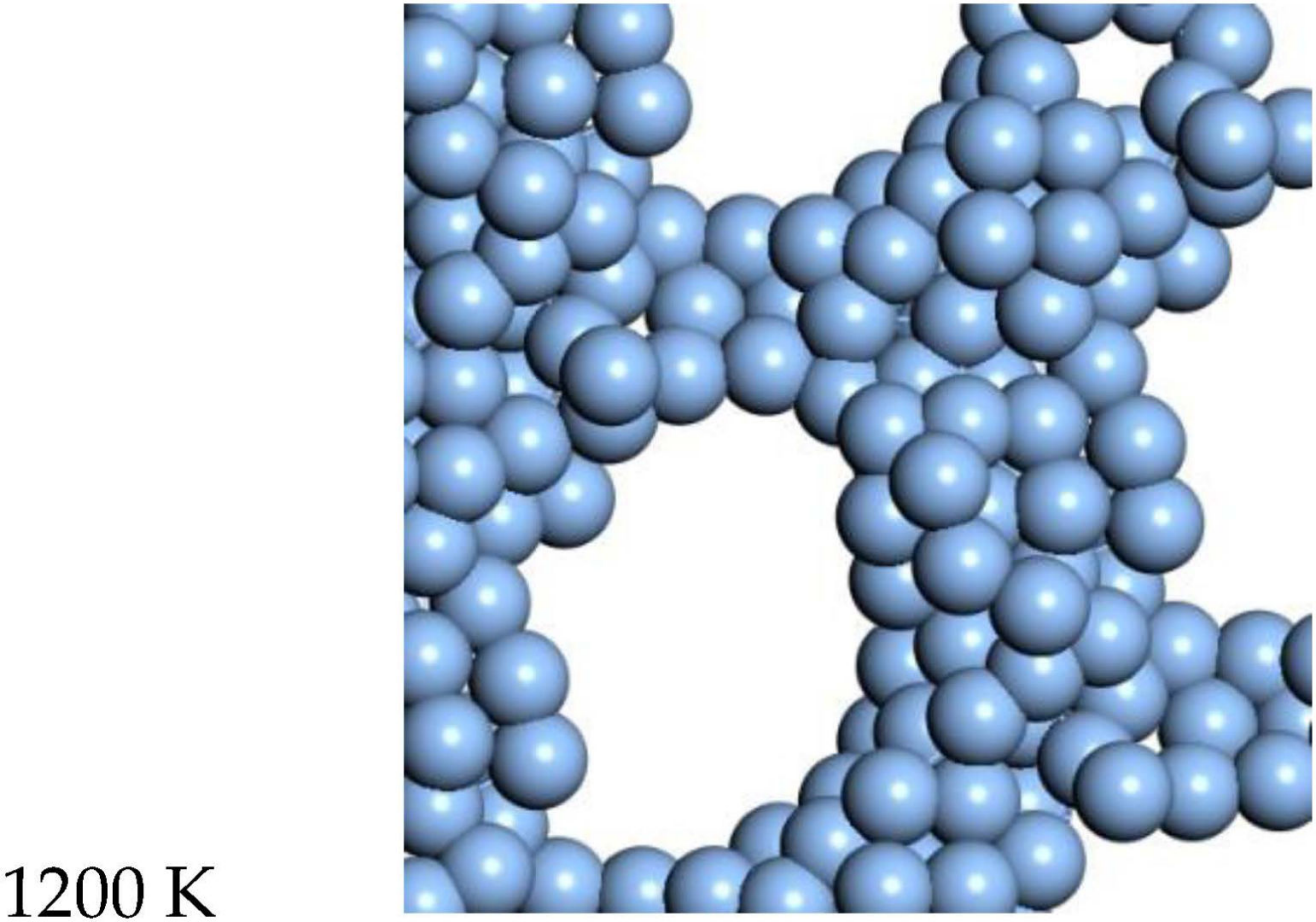
Snapshots of quasi-2D Ag layer at 1050 K and 1200 K.

For Ag, the quasi-2D liquid regime is near 1050 K. Snapshots from this temperature are shown in Figure 8, and a movie is shown in the SI. At 1200 K, we observe the conversion of the quasi-2D layer to lower energy nanorod or 3D bridge structures with 3D bonding (this is the unstable regime). This behavior is similar to the formation of 1D gold nanowires by thinning in a Transmission Electron Microscope [23].

**Figure 9:**
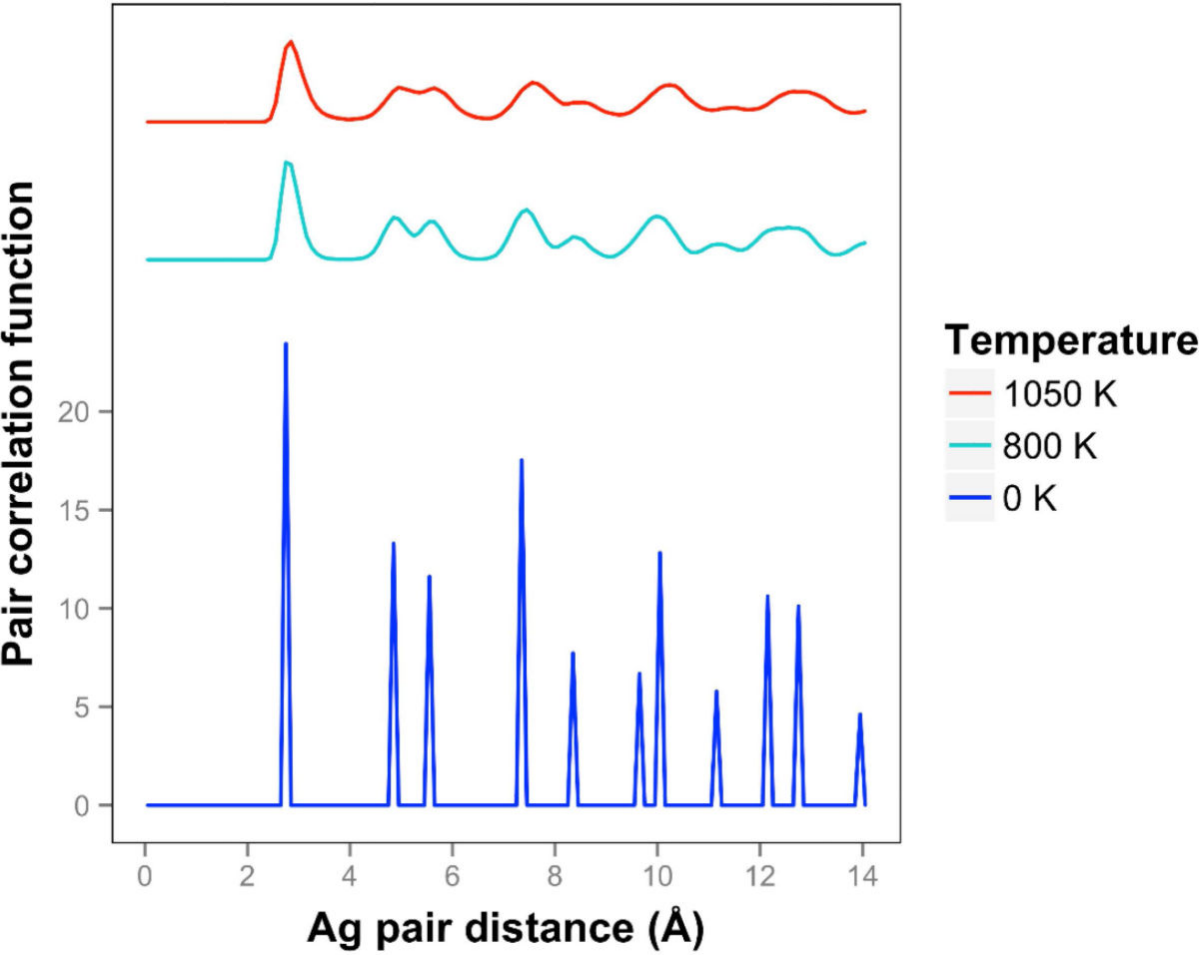
Pair correlation function for silver quasi-2D monolayers at temperatures ranging from 0 K to 1050 K. Higher temperatures are offset for visibility.

At 1050 K (where we observe quasi-2D liquid behavior in our timeframe), we see that the pair correlation function (Figure 9) is substantially reduced compared to the 800 K result. The two next nearest neighbor peaks near 5 Å are starting to come together at 1050 K, while the longer distance peaks are smoothing out and dropping away. The velocity auto correlation function for Ag at 1050 K is shown in the SI. We see that the correlations dissipate within 2.5 ps. The bond orientation order parameter for silver is shown in the SI. Strong orientation order is observed at 1050 K.

### 3.4. Copper

**Figure 10:**
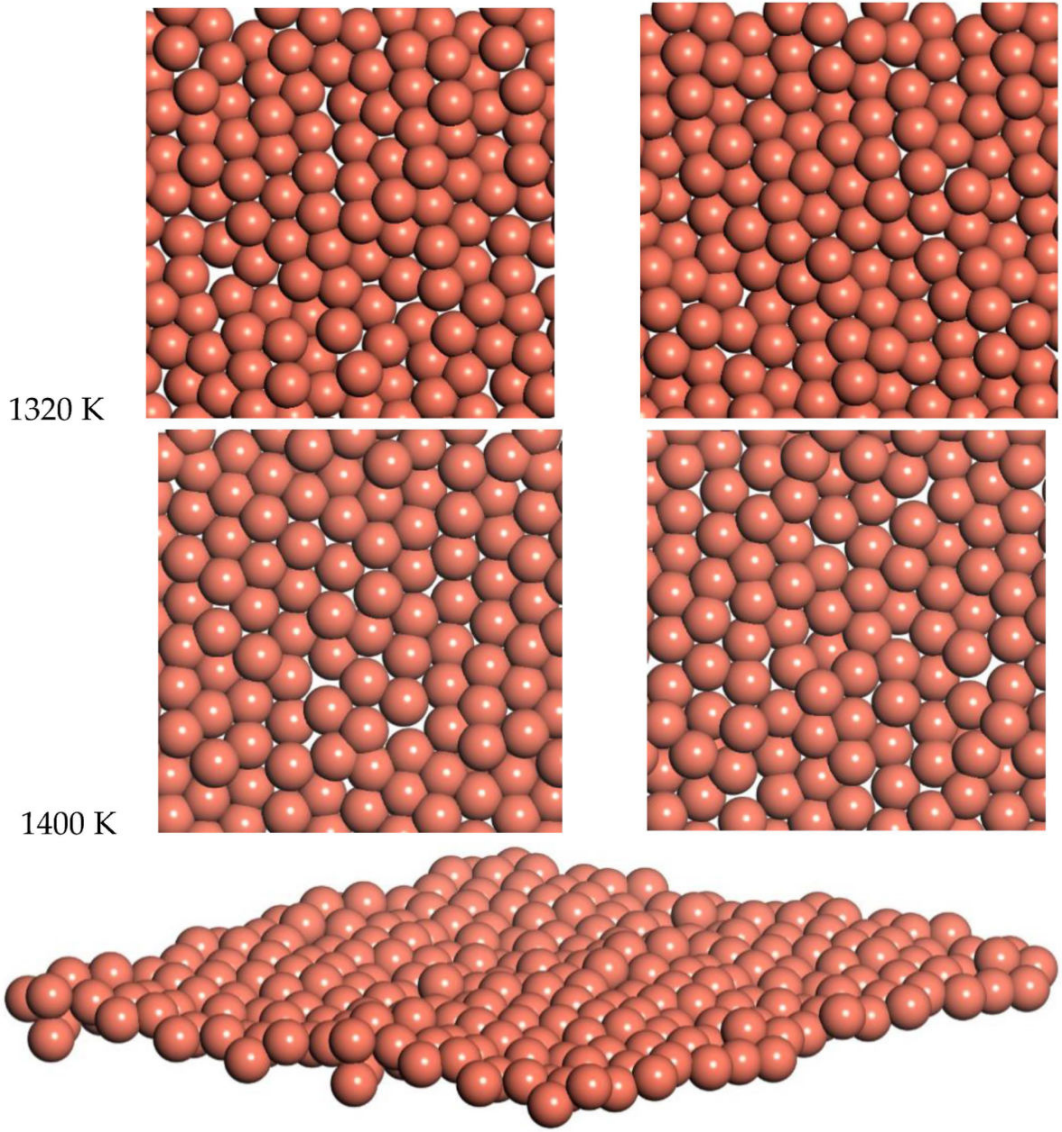
Molecular dynamics snapshot of quasi-2D Cu layer. Two snapshots separated by 0.2 ps are shown at 1320 K and 1400 K. Angle view shown at 1400 K.

Snapshots from the 1320 K and 1400 K simulations are shown in Figure 10. Slow diffusion is observed, with an RMSD of 2 Å and 1.8 Å over times of 7.2 ps and 5.5 ps for 1320 and 1400 K respectively. This leads to a prediction of the diffusion coefficient of D = 0.14 for 1320 K and D = 0.15 for 1400 K. This diffusion for the freestanding quasi-2D copper monolayer demonstrates clear quasi-2D liquid behavior. This quasi-2D liquid behavior can be observed in a movie of the 1400 K simulation in the SI.

**Figure 11:**
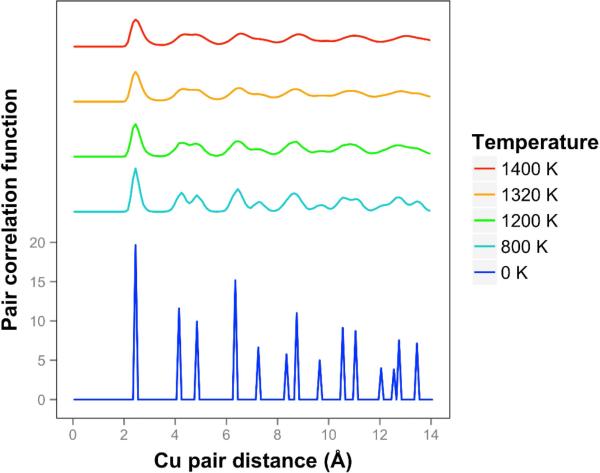
Pair correlation function for freestanding copper quasi-2D monolayers at temperatures ranging from 0 K to 1400 K. Higher temperatures are offset for visibility.

The pair correlation function for copper is shown in Figure 11 and is consistent with quasi-2D melting at the higher temperatures of 1320 and 1400 K. We see that two next nearest neighbor peaks between 4 and 5 Å merge together at 1320 K. The average number of bonds for Cu at 1400 K is 6.1, slightly higher than the 0 K value due to the presence of some 3D bonds.

One can compare the temperature ranges for the freestanding quasi-2D monolayers to the bulk melting points for these elements. We compare these results in Table S3. We find that the temperature ranges are relatively close to the bulk melting points with variations of −3%, −15, 5%, and 13%, respectively, for Cu, Ag, Au, and Pt. In particular, the Pt monolayer is stable up to very high temperatures.

We have used the highly reliable DFT-D *ab initio* method to study these systems. We feel that it is important to set a baseline using a reliable theory so that future work to extend the size or length of the simulations has a sound starting point. This work could be extended to other materials, or alloys. Our careful baseline study is not long enough or large enough to answer the question whether hexatic phase or multiple liquid phases exist in these systems, and so we will leave these questions for future studies.

## 4. Conclusions

In summary, we have seen that platinum, silver, gold, and copper can form stable freestanding quasi-2D liquid layers in freestanding monolayers at 2300–2400 K, 1050 K, 1600 K, and 1320–1400 K, respectively. Planarizing forces stabilize the quasi-2D layer. The use of high quality DFT-D theory provides a substantial temperature correction of 20% compared to the previous calculations. The use of these dispersion corrections in the DFT calculation is crucial for these systems due to the very long bond length extensions observed. Remarkably, we observe a 4% reduction in the Pt maximum bond length location as the system enters the liquid state at 2400 K. We also see a substantial change in the spring constant from 4.2 to 2.8 eV/Å^2^ for Pt as it goes into the liquid state. Pair correlation functions and diffusion measurements reveal liquid behavior in specific temperature ranges. The pair correlation function drops in the liquid state, while orientation order is reduced to a lesser degree.

## Supplementary Material

Ag Video

Au Video

Cu Video

Pt Video

Supplemental
