# Supplementary material for: Computational Study of Quasi-2D Liquid State in Free Standing Platinum, Silver, Gold, and Copper Monolayers
Source: Condens Matter. Author manuscript; Available in PMC 2017 Sep 13. (PMC5597059; doi:10.3390/condmat1010001)
Supplement: Supplemental [file NIHMS843679-supplement-Supplemental.pdf]

condensed matter

MDPI

Supplementary Material: Computational Study of Quasi-2D Liquid State in Free Standing Platinum, Silver, Gold, and Copper Monolayers

In Figures S1–S5, we show additional images of the molecular dynamics simulations. For Cu, Ag, Au, and Pt; the in-plane length of the unit cell was 19.3 Å, 22.3 Å, 21.8 Å, and 21.0 Å, respectively.

We note that CASTEP had some difficulties with some of the 2-D gold simulations. In particular, starting from the 0 K perfectly flat case, CASTEP was often unable to raise the temperature properly, and the simulations could get trapped at 0 K. For this reason, it was necessary to move some of the gold atoms in the Z direction by approximately 0.001 Å. This was sufficient to get the simulation started and the temperature controls to work better. However, for 4 fs step size, the simulations would sometimes run, but would also sometimes freeze up for up to 0.5 ps. Therefore, most of the gold simulations were run at 2.5 femtoseconds step size.

Due to the variety of step sizes and skip times for the different simulations, we include a chart shown in Table S1 with the simulation details. In general, the times were 2.5 femtoseconds or 4 femtoseconds for each material although there were some exceptions. Figures S7–S9 and Table S2 show the RMSD, and diffusion coefficient results.

There are also movie files for platinum at 2400 K, gold at 1600 K, silver at 1050 K, and copper at 1400 K. These files have duration 5 ps, 4 ps, 5.6 ps, and 3 ps respectively, and can be viewed online.

We've also included some velocity auto correlation results, some bond orientational correlation results, and bond length distribution results. (Figures S9 through S13).

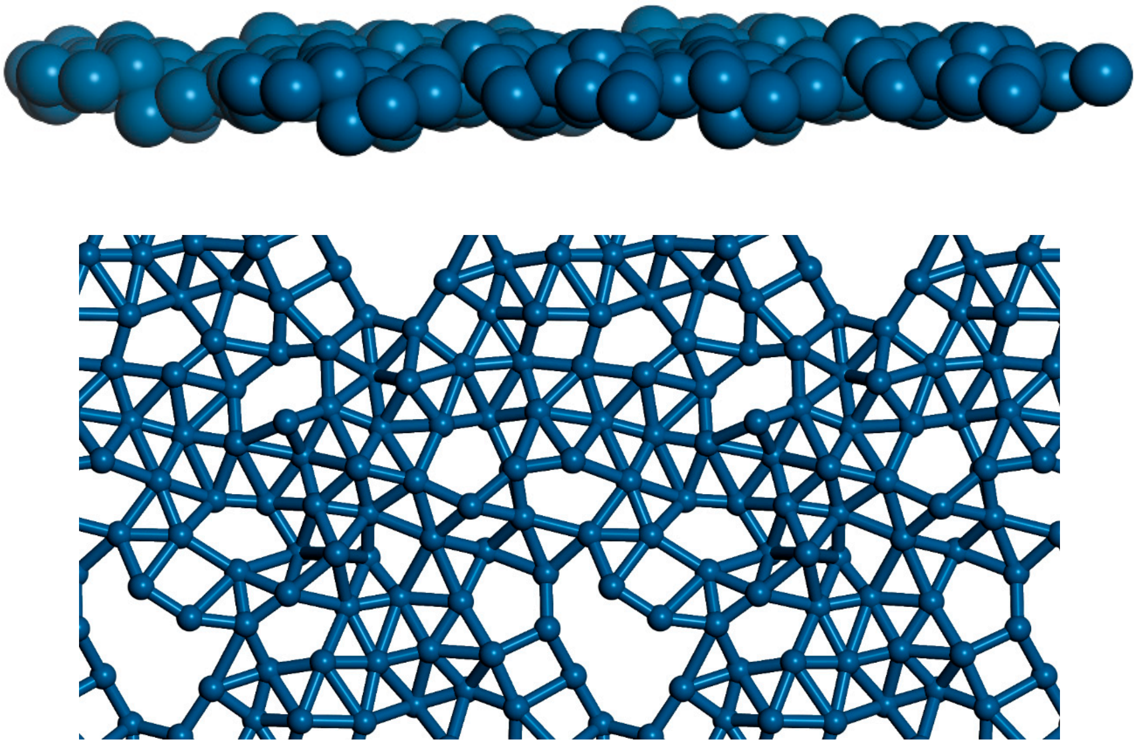

Figure S1. Platinum 2400 K side view, and ball and stick model.

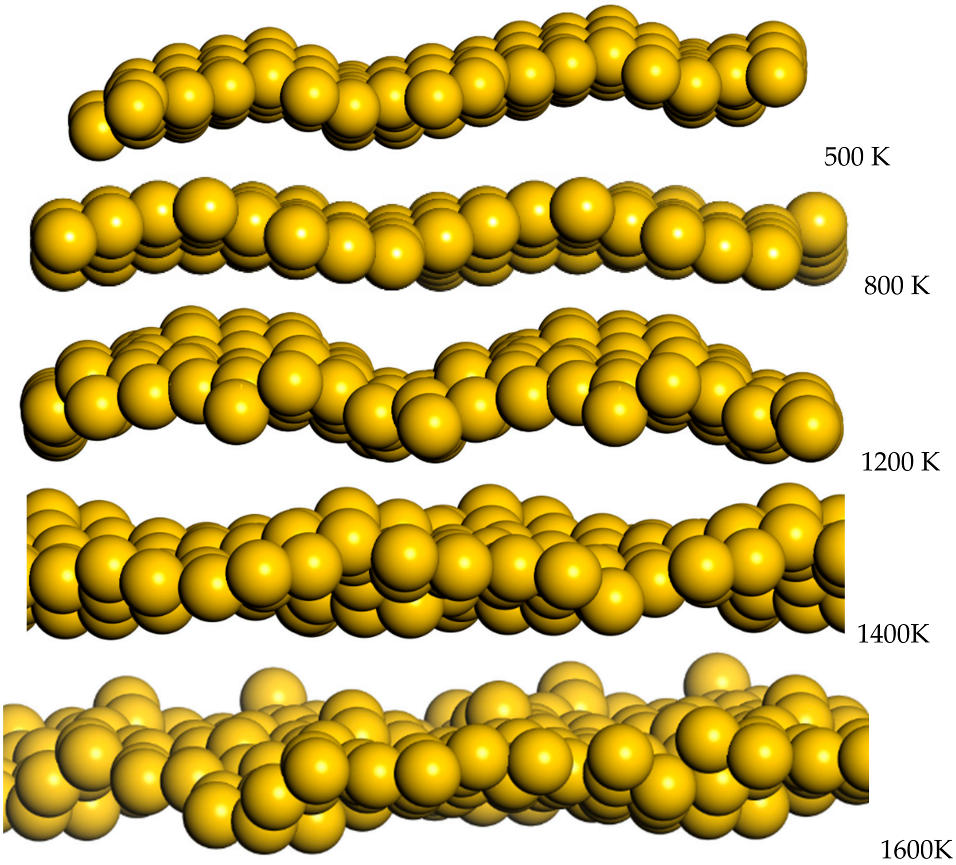

Figure S2. Side views of Au models at 500, 800, 1200, 1400, and 1600 K.

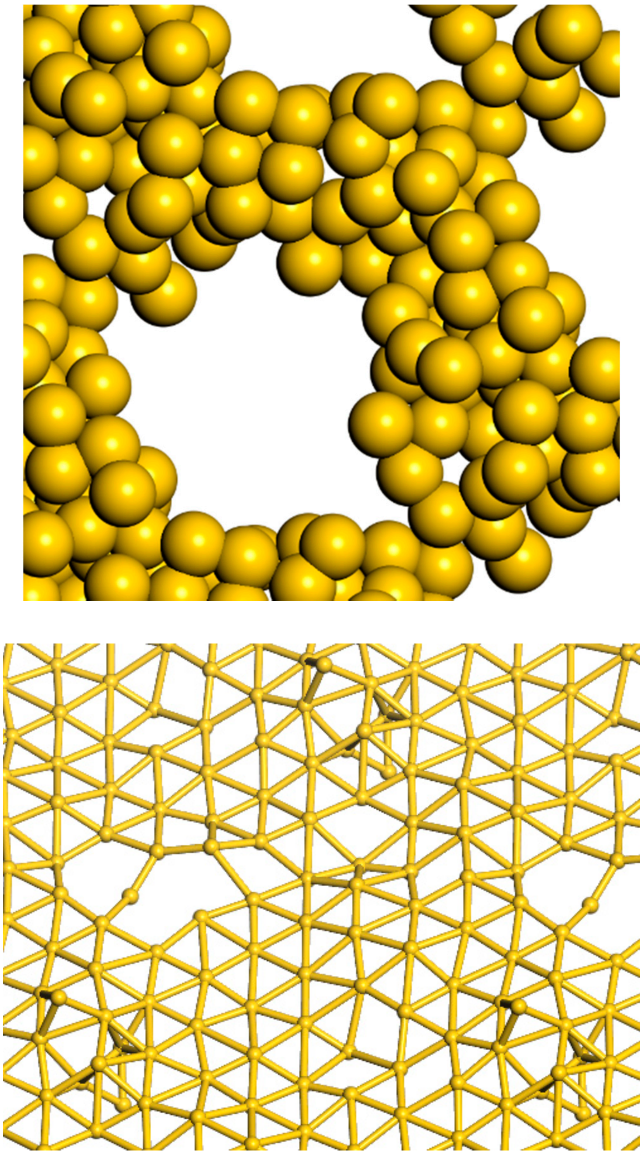

Figure S3. Last frame of gold simulation at 2000 K, top. Ball and stick model of gold at 1600 K bottom.

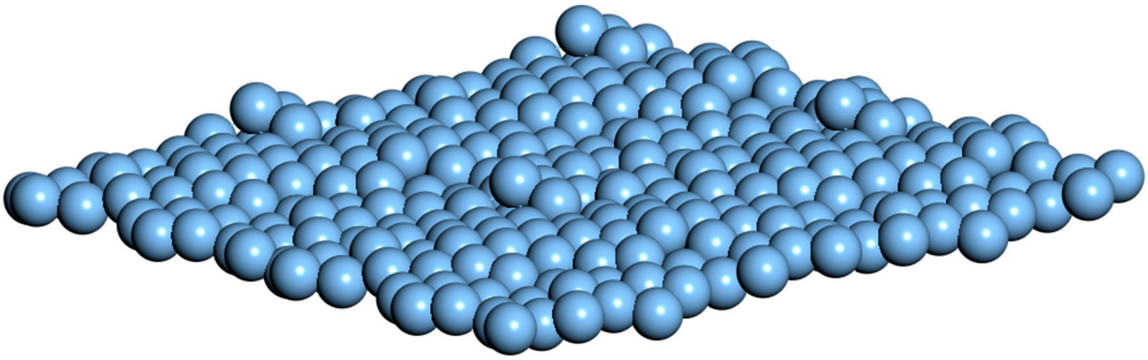

Figure S4. Silver 1050 K angle view.

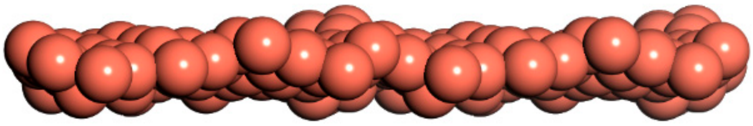

Figure S5. Copper side view at 1400 K.

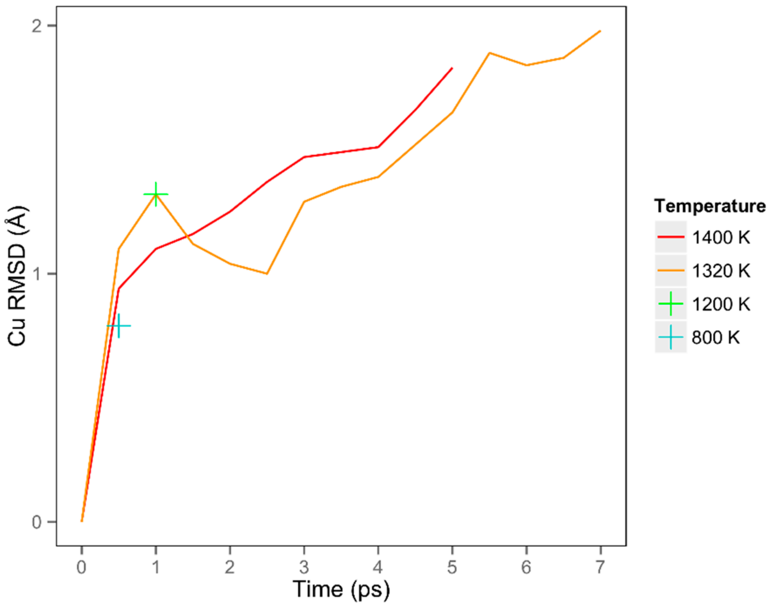

Figure S6. Plot of copper root mean square displacement versus time.

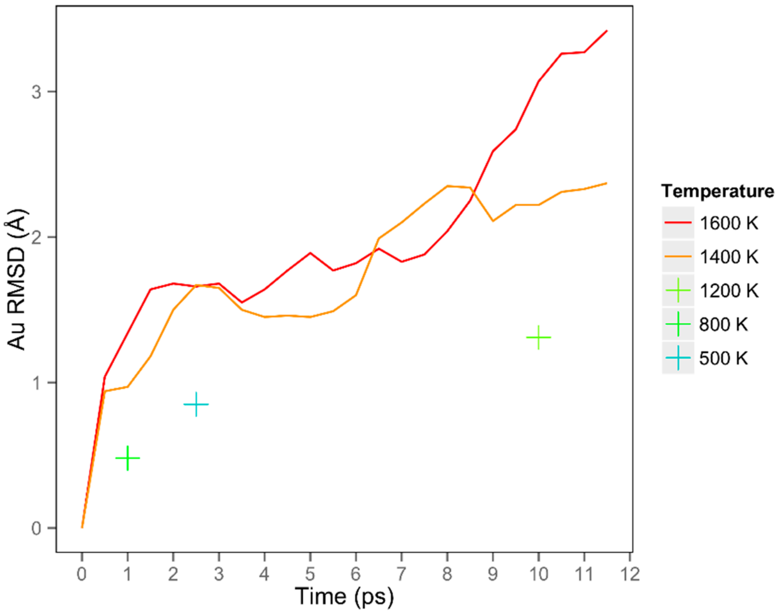

Figure S7. Plot of Au root mean square displacement versus time.

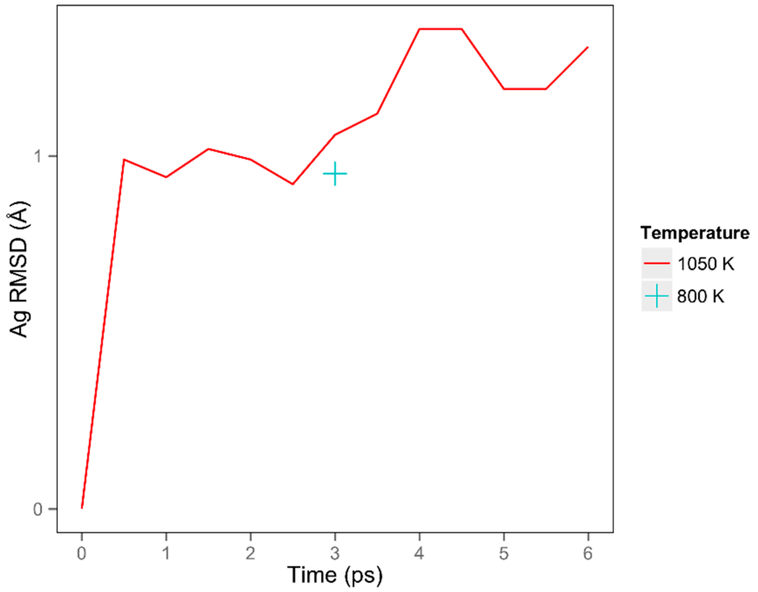

Figure S8. Plot of Ag root mean square displacement versus time.

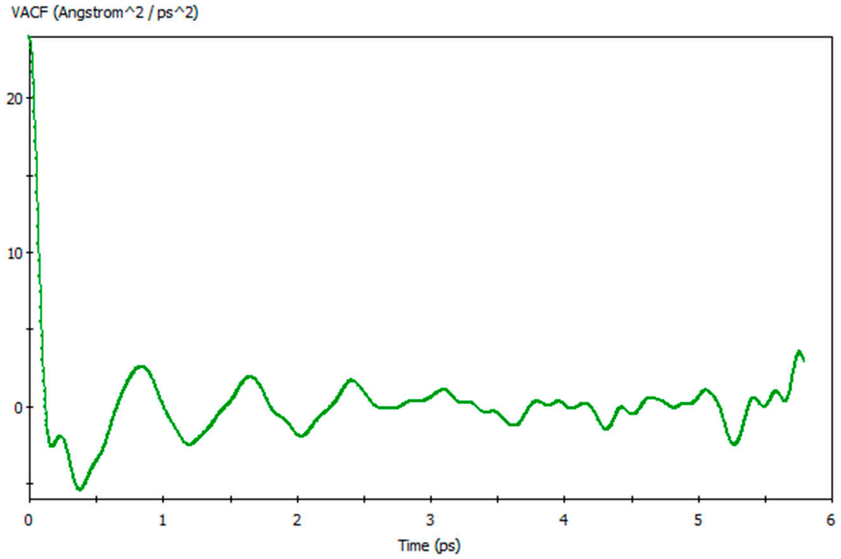

Figure S9. Plot of Ag velocity auto correlation function at 1050 K.

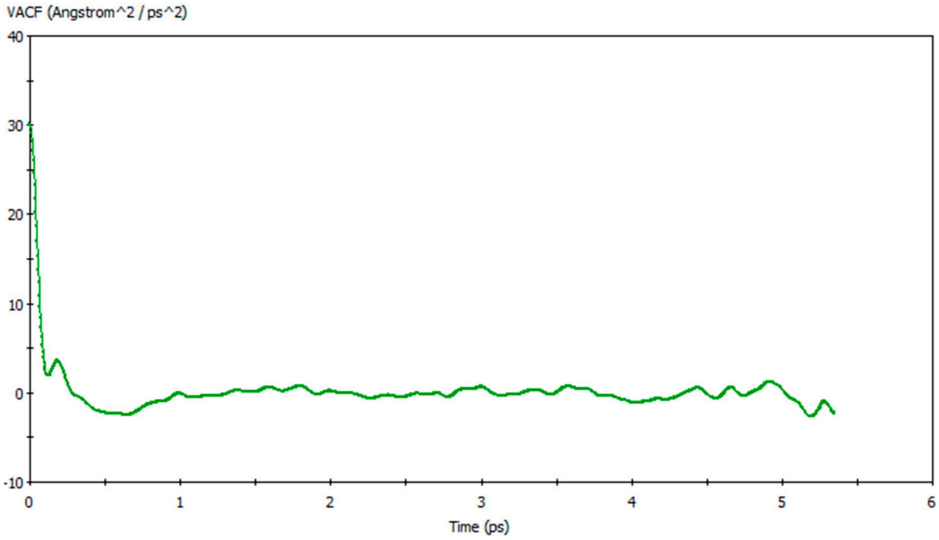

Figure S10. Plot of Pt velocity auto correlation function at 2400 K.

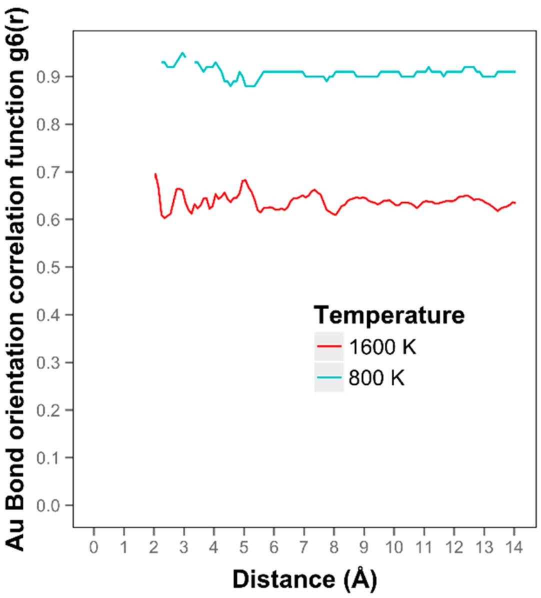

Figure S11. Orientational correlation function g6(r) for Au at various temperatures.

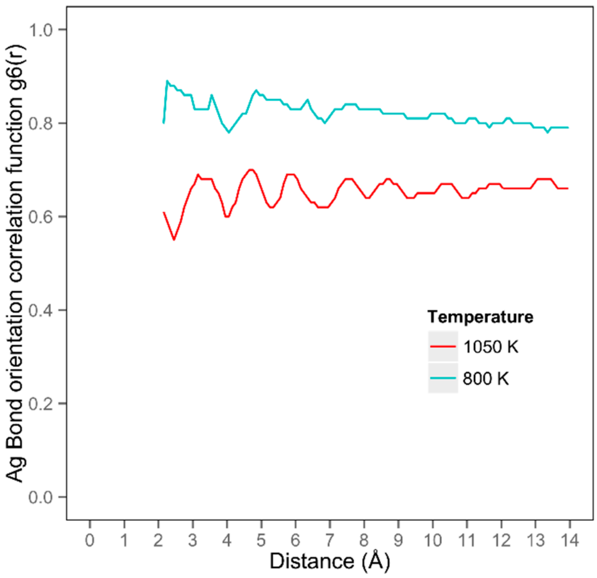

Figure S12. Orientational correlation function g6(r) for Ag at various temperatures.

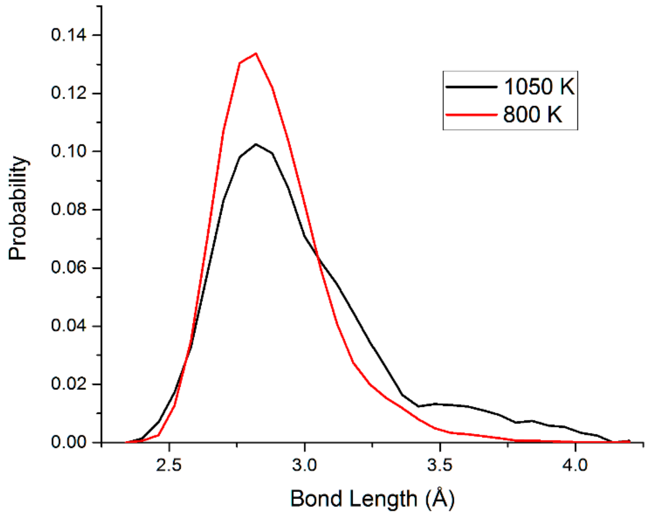

Figure S13. Bond length distribution for silver at 800 K and 1050 K.

Table S1. Simulation times and skip times for MD runs.

Cu	Temp (K)	800	1200	1320	1400
	Time (ps)	4.8	6.0	17.2	15.5
	Skip (ps)	4	5	10	10
	Step (fs)	2.5	4	2.5*	2.5*

Ag	Temp (K)	800	1050
	Time (ps)	13	18
	Skip	10	12
	Step (fs)	4	4

Au	Temp (K)	500	800	1200	1400	1600
	Time (ps)	10.7	9.2	22.0	23.7	27.8
	Skip	8	8	12	12	16
	Step (fs)	2.5	4	2.5	2.5	2.5

Pt	Temp (K)	1200	1800	2300	2400
	Time (ps)	9.10	10.00	32.00	17.00
	Skip	8	8	12	12
	Step (fs)	4	4	4	4

* The first 12 ps were run at 4 fs/step (Cu 1320 and 1400 K).

Table S2. RMSD and Diffusion Constant D Values after Equilibration.

**Cu**	Temp (K)	**800**	**1200**	**1320**	**1400**	
	Time (ps)	0.5	1.0	7.0	5.5	
	Step (fs)	2.5	4	2.5	2.5	
	RMSD (Å)	**0.8**	**1.3**	**2.0**	**1.8**	
	D			**0.14**	**0.15**	
**Ag**	Temp (K)	**800**	**1050**			
	Time (ps)	3	6			
	Step (fs)	4	4			
	RMSD (Å)	**0.9**	**1.3**			
	D					
**Au**	Temp (K)	**500**	**800**	**1200**	**1400**	**1600**
	Time (ps)	2.5	1.0	10.0	11.5	11.5
	Step (fs)	2.5	4	2.5	2.5	2.5
	RMSD (Å)	**0.8**	**0.5**	**1.3**	**2.4**	**3.4**
	D			**0.043**	**0.12**	**0.25**
**Pt**	Temp (K)	**1200**	**1800**	**2300**	**2400**	
	Time (ps)	11.0	9.4	20.0	5.0	
	Step (fs)	4	4	4	4	
	RMSD (Å)	**1.1**	**0.9**	**5.7**	**5.0**	
	D			0.40	1.27	

Table S3. Comparison of 2D Liquid Range to Bulk Melting Points for all materials.

	Cu	Ag	Au	Pt
**Bulk Melting Point**	1356	1235	1338	2041
**2D FS Liquid Range**	1320–1400	1050	1400–1600	2300–2400
**% Change**	−3%	−15%	5%	13%
